# Convolutional Neural Network-Based Cross-Media Semantic Matching and User Adaptive Satisfaction Analysis Model

**DOI:** 10.1155/2022/4244675

**Published:** 2022-04-30

**Authors:** Lanlan Jiang

**Affiliations:** Institute of Marxism and Research, Jiangxi Police College, Nanchang, Jiangxi 330000, China

## Abstract

In this paper, an in-depth study of cross-media semantic matching and user adaptive satisfaction analysis model is carried out based on the convolutional neural network. Based on the existing convolutional neural network, this paper uses rich information. The spatial correlation of cross-media semantic matching further improves the classification accuracy of hyperspectral images and reduces the classification time under user adaptive satisfaction complexity. Aiming at the problem that it is difficult for the current hyperspectral image classification method based on convolutional neural network to capture the spatial pose characteristics of objects, the problem is that principal component analysis ignores some vital information when retaining a few components. This paper proposes a polymorphism based on extension Attribute Profile Feature (EMAP) Stereo Capsule Network Model for Hyperspectral Image Classification. To ensure the model has good generalization performance, a new remote sensing image Pan sharpening algorithm based on convolutional neural network is proposed, which increases the model's width to extract the feature information of the image and uses dilated instead of traditional convolution. The experimental results show that the algorithm has good generalization while ensuring self-adaptive satisfaction.

## 1. Introduction

With the popularity and development of the global network, data such as text, images, and videos from various social networking sites, news sites, and mobile apps are in explosive growth every day. Web data affects entire society on a large scale and profound level, influencing how countless people think, express, and behave. Unlike traditional structured and unstructured data, web data is represented in various forms such as text, images, audio, video, etc. These different types of media data obtained from other channels are combined to define comprehensive knowledge and describe objective facts in a new form with multiple perspectives and depth [1]. This way of presenting information is called “cross-media”. The richness of details in cross-media itself provides unprecedented development opportunities for the new generation of multimedia semantic mining and brings new challenges for cross-media-related research [2]. By analyzing the user satisfaction scores of current search results and the number of selected recommendation words, we explore the relationship between user adaptive satisfaction and user selection of semantic matching recommendation function under different categories of query words; by analyzing the user satisfaction scores of current semantic matching results and the novelty scores of selected recommendation words, we explore the relationship between user satisfaction and user selection of query novelty under different categories of semantic matching task. The relationship between user satisfaction and the wonder of user-selected queries under various semantic matching jobs is explored. Finally, by analyzing user log information, user satisfaction with the current semantic matching result is reflected by user clicking and page-turning behaviors. An adaptive query recommendation model is proposed by integrating user satisfaction with the recent semantic matching result. Initially, many research works were devoted to semantic mining of single media and used it for media retrieval, which is characterized by retrieval results and user query samples belonging to the same media category, such as text retrieval. Because cross-media data has cross-modal and cross-platform properties, it is necessary to analyze the multimedia content itself and at the same time reasonably use the association relationship of the data itself to obtain more profound and more accurate cross-media content understanding [3]. When multiple media coexist and express the same subject matter, it is most common for text and images to appear together. For example, the need for these two media to be used as mutual search results is most common in daily searches. This kind of retrieval does not require correlation relationships between different media types.

Large-scale multimedia data are usually strongly correlated in terms of high-level semantics. Cross-media data refers to those data that express semantically similar content but appear in different modalities, different sources, different contexts, etc. Cross-media data are usually characterized by cross-modality, cross-source, and cross-space. Cross-modality means that cross-media data express the same concept, the same semantics, or the same event through different modalities such as image, video, and text. Cross-source refers to cross-media data from other sources but expresses similar semantics [4]. Cross-space refers to the data that coexist in information space and the physical world with the help of interaction behavior mechanisms. Taking advantage of the correlation between cross-media data and digging deeper into the semantics of the information contained in the data is vital for people's travel, life, and society's development. These data have correlation and multimedia characteristics, which reveal the mechanism of coupling mapping and mutual influence between information space, physical world, and human society under the interaction of human information, and realize the crossing of information space and physical space [5]. Taking advantage of the correlation between cross-media data and deeply mining the information semantics contained in the data are of great significance to people's travel, life, and the development of society. By analyzing cross-media data such as pictures and comments in social networks, we can effectively control some current hot tourist destinations and predict the problems affecting tourism safety, which can help tourism management departments or related organizations to formulate corresponding response strategies. By analyzing, learning, and recognizing the semantics of these cross-media data in the tourism field, we can effectively monitor the safety of tourism scenes. Semantic learning based on cross-media tourism data for identification and monitoring effectively achieves tourism safety.

The paper on data dimensionality reduction using neural networks has stimulated the interest of a wide range of scholars. Later, it was gradually applied to the field of fault diagnosis by scholars, and mechanical fault detection based on deep learning aims to achieve a nonlinear relationship between the extracted features and faults by deep convolutional networks for autonomous extraction of depth-invariant features from the data [6]. For cross-media retrieval, it does not rely on features such as shape, size, sound, etc. of media objects to discern whether media information is like each other, but the semantic information expressed by these media objects to determine the similarity between them, and the challenge here is how to unify media data of different modalities to the same semantic level, which is not available in traditional retrieval techniques. To a certain extent, it solves the background of people relying on specialized domain knowledge, and this automatic feature extraction approach obtains end-to-end mechanical fault diagnosis. With the development of GPU, the classification capability and feature extraction speed of deep convolutional neural nets have been substantially improved. In recent years, deep learning has been successfully applied in face recognition, computer vision, sentiment analysis, and fault diagnosis due to the advantage of fusing feature extraction and classification into one framework. Therefore, the use of deep learning for depth-sensitive feature extraction of different bolt loosening to achieve accurate diagnosis of bolt loosening faults has broad application prospects in engineering applications [7]. The relationship between user adaptive satisfaction and user selection of semantic matching recommendation function under different categories of query words is explored by analyzing the user satisfaction score of current search results and the number of selected recommendation words; the relationship between user satisfaction and user selection of query under different categories of semantic matching task is explored by analyzing the user satisfaction score of current semantic matching results and the novelty score of selected recommendation words. Convolutional neural networks introduce convolutional and pooling operations, which can fully extract the local spatial semantic features of images, and pooling operations can expand the perceptual field and extract more advanced image features to achieve image recognition. The relationship between user satisfaction and the novelty of user-selected queries under different semantic matching tasks is explored. Finally, by analyzing user log information, user satisfaction with the current semantic matching result is reflected by user clicking and page-turning behaviors. An adaptive query recommendation model is proposed by integrating user satisfaction with the recent semantic matching result.

## 2. Related Works

With the rapid development of deep learning, modeling text data using deep neural networks becomes standard. The self-encoding algorithm is the first method applied to feature extraction of text data. The core idea of the self-encoding algorithm is to design the input and output of the neural network with the same number of neurons, use the input data as the output correction data, and use the low-dimensional vector of the intermediate layer to represent the feature vector of the text [8]. An algorithm for feature extraction based on deep noise autoencoder is proposed for short text semantic sparsity and multiple meanings, using L-norm1 and deep self-coding for text feature extraction, which makes the algorithm improve significantly in clustering effect. A quick text feature extraction algorithm based on stacked noise-reducing autoencoders is proposed, extracting richer semantic information by deepening the depth of the self-coding algorithm model and stacking multiple self-coders. A convolutional self-coding text feature extraction algorithm is proposed, which combines the self-coding algorithm with a convolutional neural network, and uses the intermediate results of the self-coding algorithm as the input of the convolutional neural network to uncover the syntactic and semantic structures in the text [9]. A deep sparse self-encoder-based algorithm for sentence semantic feature extraction is proposed, which uses the self-encoder unsupervised learning of the nonlinear features of the text, maps the high-dimensional light input to low-dimensional dense semantic features, and obtains relatively effective results on the clustering task.

With the rapid development of technology and the Internet, the accumulation of data will become unpredictable, and the visualization and analysis of the data after mining have become especially important. The potential correlations between data and the implied evolutionary laws can be seen [10]. Data visualization includes knowledge visualization, information visualization, scientific visualization, information graphics, and visual design. Compared with fully connected neural networks, the parameter size of convolutional neural networks is significantly reduced, which can avoid the overfitting problem and enhance the training depth of the network, thus improving the classification function of neural networks for images. For the above data visualization methods, with other appropriate organization and collation, to some extent, the hidden intrinsic correlations or implied meanings between the data can be observed. Tables, graphs, hotspot diagrams, and even text, whether static visualization of dynamic visualization, can explore the root of the problem, find the answer to the question, and discover the hidden relationship between the data through appropriate methods and means. Currently, in the field of ample data research, data visualization is generally applied to the visual representation of large-scale nonnumerical information resources.

Xu et al. propose an integrated learning-based hash code learning strategy. Multiple “weak hash functions” and their weights can be learned, and the weighted Hamming distance across media can be calculated [11]. The graph representation-based approach uses a unified graph structure to represent similarity information within media and similarity information between media [12]. The feature space corresponding to the minor feature of this graph representation is the cross-media subspace found. Xu et al. proposed a graph decomposition method based on intra- and intermedia relationship modeling for cross-media hash learning based on a similar idea [13]. With the development of deep understanding and its remarkable achievements in image classification, people have also started to apply deep networks to cross-media semantic mining [14]. In addition to the deep typical correlation analysis mentioned above, Li and Yuan proposed a deep semantic matching method using convolutional neural networks and fully connected networks to map images and text to label vectors in 2017, which is a leading method in terms of accuracy [15].

## 3. Convolutional Neural Network-Based Cross-Media Semantic Matching and User Adaptive Satisfaction Analysis Model Design

### 3.1. Convolutional Neural Network Model Construction

**Figure 1:**
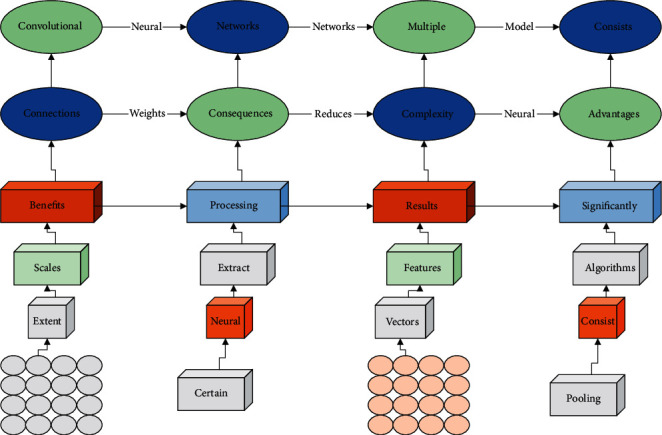
Convolutional neural network structure diagram.

Convolutional neural networks are composed of multiple two-dimensional planes; each consists of various neurons. Convolutional neural networks use local connections and shared weights to reduce the number of consequences, which effectively reduces the complexity of the network model. Based on these advantages, convolutional neural networks have many benefits in dealing with image problems, and many image processing models based on convolutional neural networks have been proposed. The results achieved by them are significantly improved compared with traditional algorithms. For example, Gogo Le Net is a famous classical network in image recognition, by using different sizes of convolutional kernels to change the perceptual field of the convolutional kernels in the web and finally stitching the features of different scales together to form the final one. The traditional neural network uses multiple nonlinear activation functions to extract features in layers by composite splicing, and the network automatically learns features during the training process, which reduces the workload of manual feature extraction to a certain extent. However, the standard neural network features are modeled as one-dimensional vectors, which destroy the spatial information of the features. For this reason, scholars have proposed convolutional neural networks. Convolutional neural networks introduce convolutional and pooling operations, which can fully extract images' local spatial semantic features. Pooling operations can expand the perceptual field and extract higher-level image features for image recognition. Convolutional neural networks usually consist of an input layer, a convolutional layer, a pooling layer, and a fully connected layer. The structure of a convolutional neural network is shown in Figure 1. Deep learning has been successfully applied in face recognition, computer vision, sentiment analysis, and fault diagnosis due to fusing feature extraction and classification into a single framework.

The convolutional layer plays a key influential role in the CNN network structure, enabling feature extraction from the input image. As the first step of CNN, it tries to reduce and capture helpful information by using the same kernel window to slide the whole picture to detect (convolve) specific image patterns to reduce the number of parameters (convolutional kernel weights). In addition, it uses multiple convolution kernels to extract image features in various ways [16]. One of the kernels scans the input in specific steps and padding to store them by convolution into a mapped feature map as input for the next layer, which acquires the input image and computes the output neurons connected to the local regions of the information. Each part performs a dot product using its respective weights. In this case, the feature map formed by the convolution kernel of the *k* layer is defined as *z*_*i*_^*k*^, the weight matrix is defined as *w*_*i*_^*k*^, the bias is defined as *b*_*i*_^*k*^, and the computational equation is given as

(1) $$\begin{array}{c} Z=f\left(W_{i}^{k}\times {Ζ}_{i+1}^{k}-b_{i}^{k}\right). \end{array}$$

The function of the pooling layer is to compress the input feature map and extract the main features, making the feature map smaller and simplifying the computational network complexity. This layer aims to remove irrelevant information and keep only relevant information. The input of this layer is the output of the nonlinear layer. The result of this layer is a simplified version of the input. The coating has pool cells organized topographically and connected to local regions in the information from the nonlinear layer. The pooling layer performs a form of nonlinear downsampling to reduce further the number of parameters, computations, and input noise. It divides the input into a set of nonoverlapping blocks and samples one element from each block, where the *i* feature map formed by the *k* convolution kernel of the layer is defined as *Z*_*i*_^*k*^, the feature map created by the convolution kernel of *k*, the *i* − 1 coating is defined as *Z*_*i*−1_^*k*^ the bias is defined as *b*_*i*_^*k*^, and the preset downsampling style is down, and the computational equation is given as

(2) $$\begin{array}{c} Z_{i}^{k}=f\left[\text{down}\left(\frac{\sqrt{Z_{i-1}^{k}+1}}{b_{i}^{k}}\right)\right]. \end{array}$$

In a fully connected layer (FCL), each neuron is connected to all neurons in the upper layer. Convolutional neural networks output targets as classes after multiple convolutions and pooling layers, so a fully connected layer is needed to map the output feature vectors to types. Deep typical correlation analysis considers the correlation between different media types and taps this correlation to a great extent through nonlinear mapping, but it does not consider the semantic information of various media types themselves, and directly ignoring the semantic information will lead to less precise intermedia relationships in the common subspace. The fully connected layer can expand the previous layer's output into a column vector that integrates local information with class distinction in the convolutional or pooling layer. This layer acts as a classifier in the whole convolutional neural network. This paper chooses the SoftMax classifier, a supervised learning algorithm with the primary category of input samples. The same selection cannot belong to more than one category simultaneously, suitable for solving multiple classification problems, where ∂_*j*_^*l*+1^ denotes the output value of the first *i* neuron in the first *l*+1 layer; denotes the weight between the first *i* neuron in the *w*_*ij*_^*l*^*l* layer and the *l*+1 the first neuron in the coating *jp*_*j*_^*l*^(*i*) denotes the corresponding value of the neuron in the first *l* layer of the pooling operation; *b* denotes the bias. Many research works are devoted to semantic mining of single media and using it for media retrieval, characterized by retrieval results and samples of user queries belonging to the same media category, such as text retrieval. Since cross-media data have properties such as cross-modality and cross-platform, it is essential to analyze multimedia content and make reasonable use of the association relationship of data itself to obtain a deeper and more accurate understanding of cross-media content.

(3) $$\begin{array}{c} \partial_{j}^{l+1}=\sum_{i=1}^{n}w_{ij}^{l}P_{j+1}^{l}+b_{j-1}^{l}. \end{array}$$

The fully connected layers are in the network's last layers in the convolutional neural network structure. Each node of the fully connected layer is kept connected to the nodes of other network layers. This layer can significantly improve the parameter characteristics of the fully connected layer through convolutional operations. It also enables the integration of the local information of the pooling and convolutional layers. Softmax coating is used to identify and classify the output values of the fully connected layer by Softmax logistic regression, which maps each output value in the (0,1) range, i.e., the percentage of each value in the overall weight, to complete the classification of the output values. Softmax logistic regression is calculated as follows: the fully connected implicit layer is activated using a ReLU function that Softmax can convert the neurons of the last layer of the implicit layer:

(4) $$\begin{array}{c} \text{Softmax}=\frac{\exp\left(\partial_{j}\right)}{\sum_{n-1}\exp\left(n+1\right)}. \end{array}$$

**Figure 2:**
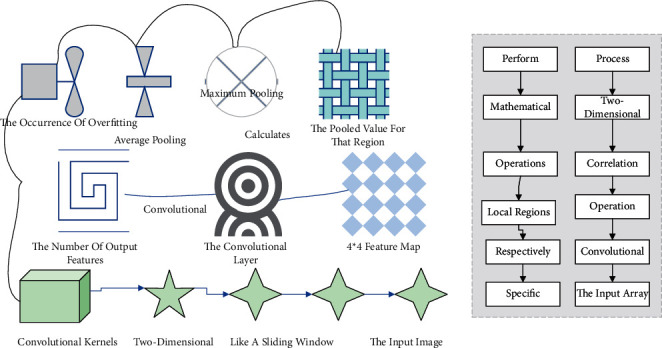
Convolutional operation process.

With the occurrence of overfitting, two standard pooling methods include average pooling and maximum pooling. Average pooling calculates the average value of an image region as the pooled value for that region. Complete pooling is to select the total value of the image region as the value after pooling for that region. For a 4 ∗ 4 feature map, after a filter with step size 2, size 2 ∗ 2, the core of the convolutional neural network is the convolutional layer, which generally consists of multiple convolutional kernels, and the number of output features of the convolutional layer is positively related to the number of convolutional kernels. The convolutional kernels are two-dimensional arrays, like a sliding window, that slide over the input image and perform mathematical operations on the local regions they pass through, respectively. The specific process is shown in Figure 2. In the two-dimensional mutual correlation operation, the convolutional window starts from the top leftmost part of the input array. Among artificial neural networks, convolutional neural networks are one of the typical applications, which can significantly reduce the number of weights and greatly simplify the network model complexity through locally connected, weight-sharing processing mechanisms. It sequentially slides over the input array from left to right and top to bottom. During the sliding process, the parameters of the input array and the corresponding positions of the convolution kernel are multiplied and accumulated. The resulting value is part of the output result of that convolution layer.

In the CNN structure, after multiple convolutional and pooling layers, 1 one or more fully connected layers are connected. Each neuron in the *N*th layer of the fully connected layer is connected to all neurons in the *N* − 1th layer, while the same layer is not connected. The fully connected layer can integrate the local information with category differentiation in the convolutional or pooling layers.

### 3.2. Cross-Media Semantic Matching and User Adaptive Satisfaction Model Design

The semantic matching algorithm (SM) does not consider the deep semantic connections between different types of media data. It completes the mapping of the joint space based only on the semantic relationships of their classes. Still taking text and image data as an example, image features and text features are extracted from the *K* original class data and mapped to the semantic space corresponding to this *K* class. A standard mapping method is multivariate logistic regression to calculate the posterior probability distribution, generating a linear classifier that can be interpreted probabilistically. Logistics regression calculates the posterior probability *x* belonging to a class *j* by substituting the data *x* into the following logistics equation:

(5) $$\begin{array}{c} P\left(v\right)=\frac{\left[Z\left(x-w\right)+1\right]}{\exp\left(w_{j}^{T-1}-1\right)}, \end{array}$$

where *Z*(*x*, *w*)=∑_*j*_exp(*w*_*j*_^*r*^*x*) is a normalization constant, *r* is the kind label, *x* is the feature vector of the input space, and *w*=(*w*_1_, *w*_2_,…, *w*_*k*_) (where *w*_*j*_ is the dimensional parameter vector of *n* corresponding kind *j*, which is also the parameter to be calculated in the logistic stiff regression process). By this method, both text features and image features in different dimensions can be mapped to the same *k* dimensional semantic space, and cross-media semantic mining can be accomplished. Simply speaking, the multimedia objects of other modalities are mapped to a homogeneous semantic subspace by semantic learning of the extracted underlying features. Then the cross-media retrieval task is accomplished by matching the similarity based on semantic relatedness. For text documents *T* and images *I*, their corresponding feature spaces are labeled as *R*_*r*_^*m*×*p*^ and *R*_*I*_^*m*×*q*^, respectively, with *m* denoting the number of samples because text documents and ideas exist in pairs. Hence, their sample numbers are the same, *p* and *q* tell the dimensionality of text document and image features, respectively, *S*_*I*_^*m*×*k*^ represents the isomorphic semantic subspaces corresponding to text documents and images after semantic mapping, *R*_*T*_^*m*×*q*^ and, *R*_*I*_^*m*×*q*^ represent the number of semantic categories, then, the semantic categories can be expressed as *v*=(*v*_1_ … *v*_*k*_). On the premise of obtaining the underlying feature space *R*_*T*_^*m*×*q*^, *R*_*I*_^*m*×*q*^ semantic mapping is performed on text documents and images, respectively. The corresponding isomorphic semantic subspaces *S*_*T*_^*m*×*k*^ and *S*_*I*_^*m*×*k*^ are independently trained from the underlying feature space. The same semantic concept base model generates both. The isomorphic semantic subspace obtained by training is the probability value of each sample belonging to its corresponding class, i.e., a probability distribution vector. This probability distribution vector can be derived from the above.

(6) $$\begin{array}{c} P_{\left(v_{i}-t\right)}=\int_{k}^{i}\sqrt{\frac{v-1}{T}}. \end{array}$$

In real search scenarios, users usually have page-turning behavior when dissatisfied with the current search results or want to get more results. In modeling user satisfaction with recent search results, the user's page-turning behavior is regarded as dissatisfaction with current search results. Then user satisfaction with the recent search results can be formally defined as *AP*(*q*_0_) where *AP*(*q*_0_) denotes the average accuracy of the query term (*q*_0_) on the current search result page; *n* is the number of pages the user has turned backward. If the user turns the page to the first three pages, it means that the user has viewed the content of the previous 3 pages; then *n* is 3. log(*n*+1) is the conversion factor, which increases with the number of pages turned by the user and thus reflects the decrease in user satisfaction. In this paper, we ignore the user's direct page-turning behavior and consider the user's page-turning behavior to turn the page to the next search result page after browsing the current search result page. After extracting text and image features using deep learning methods, the method then uses deep semantic matching methods to complete the cross-media retrieval part, simply a three-layer fully connected neural network to map the previously extracted text features and image features to a K-dimensional space.

(7) $$\begin{array}{c} SAT\left(q_{o}\right)=\frac{\sqrt{AP\left(q_{o}\right)}}{\log\left(n+1\right)}. \end{array}$$

**Figure 3:**
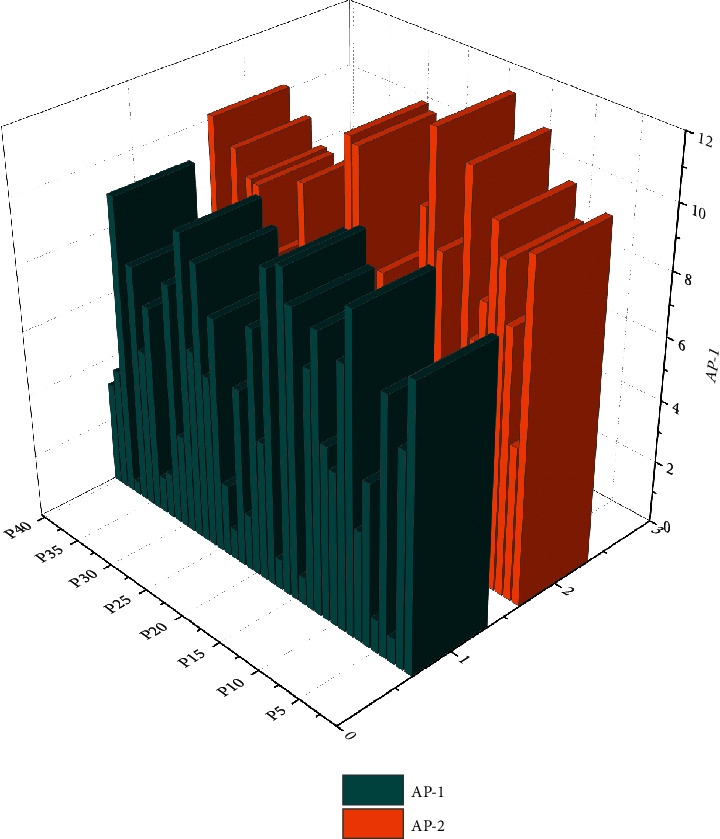
Variation of accuracy rate.

To better estimate user satisfaction with the current search results, this paper considers a correlation between user satisfaction with the recent search results and user satisfaction with the previous query [17]. Suppose there is no satisfactory click behavior and page-turning behavior in the current search query term. In that case, the average accuracy of the last term query is used to estimate the average accuracy of the current query recommendation term. For this purpose, the average precision (AP) of each query in the experimental dataset is counted in this paper. The variation of the average accuracy of adjacent queries is calculated as shown in Figure 3.

The cross-media semantic mining algorithm based on a convolutional neural network does not use the traditional feature extraction method in the image feature extraction stage. But train the convolutional neural network based on the images and tags in the Image Net database, and then fine-tune the web with relevant images. Finally, the retrieved images are used as the convolutional neural network's input, and the convolutional layer's output is calculated to select the appropriate intermediate layer for the image features. The components are first extracted once using the traditional TF-IDF or LDA method when extracting text features. Then it is used as the input of the fully connected neural network to train this network for feature extraction. After removing text and image features using deep learning methods, the method then uses deep semantic matching methods to complete the cross-media retrieval part [18]. This means that the previously extracted text features and image features are mapped to a K-dimensional space by a three-layer fully connected neural network, with K representing the total number of categories. Cross-media retrieval is completed under this semantic space based on the similarity metric, and the algorithm's effectiveness has been verified. Extracting image features by deep learning provides a new idea for cross-media semantic mining. Its excellent performance effect makes more researchers explore cross-media semantic mining techniques based on deep knowledge.

## 4. Analysis of Results

### 4.1. Convolutional Neural Network Model Analysis

**Figure 4:**
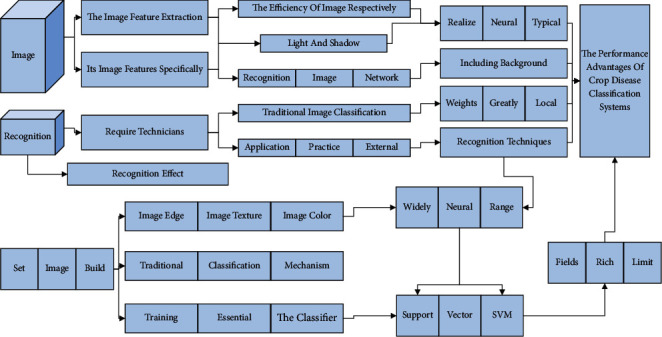
Fully connected network structure diagram.

In the traditional image classification and recognition, the image feature extraction is mainly done manually, and its image features specifically include image edge, image texture, image color, etc. According to the traditional image classification mechanism, the image features of the training set are first extracted; the essential elements complete the classifier's training. Then the test set images are classified by the classifier. A support vector machine (SVM) is one of the most widely used classifiers. In application practice, many external factors interfered with traditional image classification and recognition techniques, including background, light and shadow, etc., and require technicians with rich practical experience. Otherwise, they will significantly limit the image classification and recognition effect. In addition, cross-media semantic matching image features are relatively complex, creating great difficulty for technicians to build efficient image classification and recognition systems and to a certain extent limiting the improvement of the efficiency of image recognition. Based on the traditional image classification and recognition technology, the image classification technology based on the neural network does not need to rely on the subjective judgment of technicians. It achieves the extraction of image features with more detailed data, which can realize the robustness and completeness of image features [17]. Among artificial neural networks, convolutional neural networks are one of the typical applications, which can significantly reduce the number of weights and greatly simplify the complexity of network models through the processing mechanism of local connectivity and weight sharing. Therefore, the performance advantages of crop disease classification systems based on convolutional neural networks are more prominent and can be applied to a broader range of agricultural production fields. Among them, the operation mechanism of image classification based on a fully connected neural network is shown in Figure 4. The visualization of data mining results can reveal the potential correlation relationships and implied evolutionary patterns between data. Data visualization includes knowledge visualization, information visualization, scientific visualization, information graphics, and visual design.

In the fully connected network model, if the input image is a regular 299 × 299, the size of the input layer neurons will be as large as *n* = 299 × 299 = 89401. Therefore, the values of the input layer neurons are multiplied by the weight values, and bias values are added to obtain the importance of the hidden layer neurons. Subsequently, the hidden layer neuron values are passed through the activation function to form the input values of the neurons in the next layer. In a fully connected neural network, the weight parameters vary, and when the hidden layer contains 10000 several neurons, the scale of the hidden layer's weight parameters grows exponentially to 894011 × 0000 = 9 × 108. The fully connected neural network contains multiple hidden layers, which leads to a vast number of parameter values in the neural network model and poses a considerable challenge to network training.

**Figure 5:**
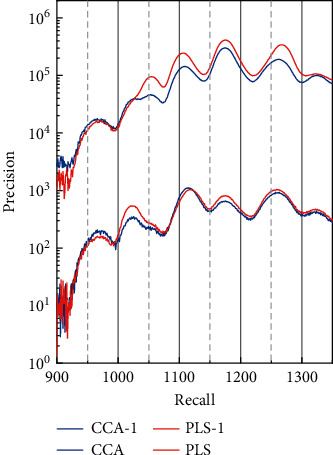
PR curves for each search method on the database.

Meanwhile, in the neural network's input layer, two-dimensional images are usually transformed into one-dimensional series, which prevents the information between pixels from being fully utilized. In addition, the number of network layers of fully connected neural networks is limited because the parameter size of fully secured neural networks is too large for network training to be carried out efficiently. However, due to its massive number of parameters, it consumes many computational resources. Therefore, the image classification technique based on the fully connected neural network has limitations in the application scope. The PR curves of each retrieval method on the database are shown in Figure 5.

**Figure 6:**
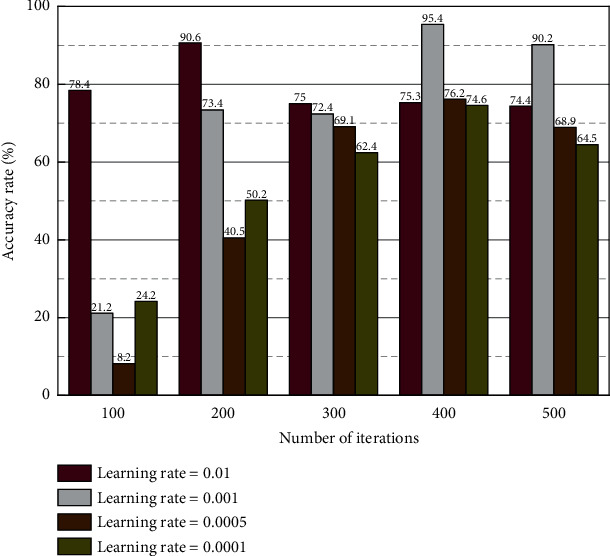
Model accuracy.

Deep typical correlation analysis considers the correlation between different media types and taps this correlation greatly through nonlinear mapping. Still, it does not feel the semantic information of various media types themselves, and directly ignoring the semantic information will lead to less accurate intermedia relationships in the common subspace. In this paper, we propose an improved deep typical correlation analysis algorithm, deep semantic typical correlation analysis (DSCCA), which maximizes the correlation of text and images using deep neural networks while ensuring semantic consistency across media types, specifically embodied by maximizing the similarity of samples in the same category for text features and image features (the variety here is not the category of media types). This is done by maximizing the similarity of samples in the same category (where the class is not the media type but the semantic category of data) and minimizing the similarity of models in different categories. The first method applied to text data feature extraction is the self-coding algorithm. The core idea of the self-coding algorithm is to design a neural network with the same number of neurons for the input and output, use the input data as the correction data for the production, and use the low-dimensional vector in the middle layer to represent the feature vector of the text. After extracting the semantic features, the Euclidean distance between pieces of the same class in images and texts is as tiny as possible. The Euclidean distance between representatives of different types is as considerable as possible in the new common subspace [19]. On the Wikipedia dataset, the average finding accuracy of the algorithm proposed in the article for text retrieval of images is 7.4% higher than that of the semantic matching algorithm, and the average finding completeness of the image retrieval of text is 7.7% higher than that of the typical correlation analysis algorithm. The category of the Wikipedia dataset is 10 and the type of the XMedia dataset is20, which makes the accuracy rate on this data slightly lower than that of the XMedia dataset. On the XMedia dataset, the average finding rate of the proposed algorithm for text retrieval of images is 6.3% higher than the average finding rate of typical correlation analysis algorithms, and the average finding rate of image retrieval of text is 7.1% higher than that of semantic matching algorithms. The model accuracy rates are shown in Figure 6.

Thus, the features of the cross-media semantic matching and user adaptive satisfaction classification mechanism based on the convolutional neural network include the following: First, based on the traditional image classification mechanism, the convolutional neural network relies more on big data to complete the image feature extraction, without the need for technicians to set up the feature extractor in advance, and the final extracted features have higher robustness. Second, compared with fully connected neural networks, the parameter size of convolutional neural networks is significantly reduced, which can avoid the overfitting problem and enhance the training depth of the network, thus improving the classification function of neural networks for images. Convolutional neural networks are introduced in this study to classify cross-media semantic matching and user adaptive satisfaction.

### 4.2. Cross-Media Semantic Matching and User Adaptive Satisfaction Model Implementation

Media information of different modalities has the same semantic meaning, which provides a strong guarantee for cross-media retrieval. For cross-media retrieval, it does not rely on the shape, size, sound, and other characteristics of media objects to identify whether they are like each other. Still, the semantic information is expressed by these media objects to determine their similarity, and the challenge here is how to unify the media data of different modalities to the same semantic level, which is not available in traditional retrieval techniques [20]. Here it also leads to another problem, i.e., the “semantic gap,” which is the difference between the underlying features and the high-level semantics due to the inconsistency between the computer-acquired media object information and the semantic information of the user's understanding of the media object, and the existence of the “semantic gap” likewise constrains the development of cross-media retrieval technology. Earlier cross-media retrieval techniques focused on analyzing the underlying features of multimedia information while ignoring the connection between the underlying features and the high-level semantic aspects of multimedia information. By analyzing cross-media data such as pictures and comments in social networks, we can effectively control some current hot tourist destinations and predict the problems affecting tourism safety, which can help tourism management or relevant organizations to formulate corresponding response strategies. This chapter examines the association between multimedia information in terms of underlying features and high-level semantics, constructs an isomorphic high-level semantic space based on the underlying feature space of different modal objects, applies the integrated learning approach to cross-media retrieval, and proposes the method of Bagging-SM to match the semantics of multimedia objects of other modalities, which has dramatically improved the accuracy of retrieval results compared with the traditional cross-media retrieval techniques.

**Figure 7:**
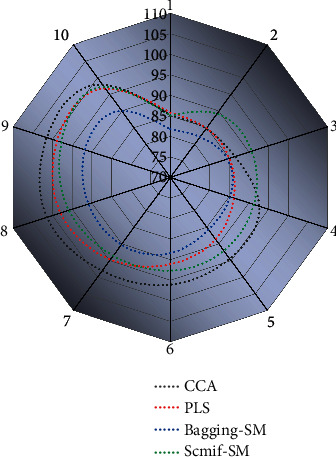
Retrieved MAP values for different categories on the dataset.

The merit of cross-media retrieval results can be judged based on these two performance evaluation metrics. A higher precision rate indicates that the algorithm is more capable of querying out relevant semantic multimedia; a higher completeness rate suggests that the algorithm is less likely to query out irrelevant semantic multimedia objects. If the relevance of the query result to the query sample is greater than the set threshold, it is considered that it should be retrieved. With “precision rate” as the vertical coordinate and “recall rate” as the horizontal coordinate, the precision rate and recall rate under different thresholds are calculated from large to small, and the corresponding curve is the PR curve by smoothly connecting these points, which can more comprehensively represent the retrieval efficiency. In general, if the PR curve of an algorithm is higher than the PR curve of another algorithm, or if the corresponding area is more extensive, we consider that the algorithm has better performance. Logistic stiff regression method, which is used to achieve cross-media semantic mining, extracts LR features of different types of media data and proposes a combined semantic space in combination with the deep semantic typical correlation analysis in Chapter 3, and introduces the solution method of integrated features. This combined feature contains both intermedia-related semantic information and intramedia semantic information. Unlike traditional structured and unstructured data, web data is represented in various forms such as text, images, audio, video, etc. These different types of media data obtained from other channels are combined to define comprehensive knowledge and describe objective facts in a new form with multiple perspectives and depths. And it can mine higher-level semantic information from cross-media data than the deep semantic typical correlation analysis algorithm. The experimental part compress the algorithm with four other mainstream cross-media retrieval algorithms on two standard cross-media datasets. It verifies the stability and effectiveness of the algorithm from different perspectives in the form of graphs. The MAP values for different retrieval categories on the dataset are shown in Figure 7.

The experimental results show that this paper's adaptive query recommendation model outperforms the benchmark model (QFG) significantly after the first day. And the model proposed in this paper performs stably with less fluctuation in *n* DCG@10 metrics. On the other hand, it increases as the size of the dataset increases. This indicates that the query recommendation model in this paper is less affected by the scale of experimental data.

**Figure 8:**
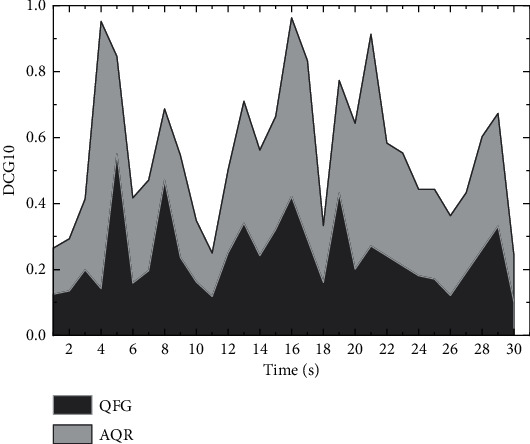
AQR and QFG experimental results.

In contrast, the query flow graph model is more affected by the scale of the observed dataset and sensitive to the scale of the experimental dataset. Large-scale multimedia data are usually strongly correlated in high-level semantics. Cross-media data express semantically similar content but appear in different modalities, sources, contexts, etc. It is possible that as the data increases, the performance of the adaptive query recommendation model in this paper will converge with that of the query flow graph model and remain stable (this hypothesis was not verified in this experiment due to the lack of sufficient data). The adaptive query recommendation model (AQR) outperforms the query flow graph model (QFG) in *n* DCG@10 evaluation metrics, and the possible reasons for this are as follows: (1) This model can select more query terms as candidate recommendation terms by exact matching and fuzzy matching, which enriches the set of candidate query terms. (2) This model can more accurately recommend candidate query terms for users by mining user search logs and integrating user satisfaction with current search results. The results of the AQR and QFG experiments are shown in Figure 8.

Cross-media semantic matching and user adaptive satisfaction analysis algorithms improve the loss function of deep typical correlation analysis. Media label information is introduced to maximize the similarity of same-category samples (the category here is not the category of media types, but the semantic category natively represented by the data) and minimize the similarity of different-category models maximum relevance. After extracting semantic features, the Euclidean distance sum of same-category pieces in images and text is as small as possible, and the Euclidean distance of different-category elements is as considerable as possible under the new common subspace. It is ensured that the learned public subspace reflects both intermedia correlation and semantic information. Finally, the effectiveness of the DSCCA algorithm is verified by doing cross-media retrieval experiments on the Wikipedia and XMedia datasets.

## 5. Conclusion

The advent of the Internet era and the rapid development of multimedia information technology have brought the convenience of information sharing and posed challenges to multimedia information services; better semantic matching and user satisfaction of multimedia information have become the main direction of research today. Cross-media semantic matching and user adaptive satisfaction are based on semantic matching of different modalities. The early cross-media semantic matching techniques focus on analyzing the underlying features of multimedia information and ignore the connection between the underlying elements and the high-level semantics of multimedia information. Analyze the association between the underlying components and the high-level semantics of multimedia information. Construct a homogeneous high-level semantic space based on the underlying feature space of different modal objects, and apply the integrated learning approach to cross-media semantic matching. The Bagging-SM approach is proposed to match the semantics of multimedia objects of other modalities. The cross-media semantic information mined by the semantic typical correlation analysis algorithm of the convolutional neural network is only at the level of similarity. Dissimilarity does not contain high-level semantic information. Therefore, this paper constructs a combined semantic space to mine higher-level semantic information. Logistic Stick regression classifies text and images separately. The classification results are first used as the respective semantic features, i.e., LR features. The extracted semantic features relate to the improved deep semantic typical correlation analysis to extract DSCCA features as the combined features. Based on the conclusions from user experiments, this paper innovatively proposes an adaptive query recommendation model that incorporates users' current semantic matching states. Under different semantic matching satisfaction states of users, other strategies should recommend matching more novel or enhanced relevant information representations for users.

## Data Availability

The data used to support the findings of this study are available from the author upon request.
